# Prediction of Type 2 Diabetes Risk and Its Effect Evaluation Based on the XGBoost Model

**DOI:** 10.3390/healthcare8030247

**Published:** 2020-07-31

**Authors:** Liyang Wang, Xiaoya Wang, Angxuan Chen, Xian Jin, Huilian Che

**Affiliations:** 1 Beijing Advanced Innovation Center for Food Nutrition and Human Health, College of Food Science and Nutritional Engineering, China Agricultural University, Beijing 100083, China; 18259800533@163.com (L.W.); 15384665858@163.com (X.W.); 2 College of Information and Electrical Engineering, China Agricultural University, Beijing 100083, China; cauapplexian@163.com; 3 College of Economics and Management, China Agricultural University, Beijing 100083, China; applexian@cau.edu.cn

**Keywords:** ensemble learning, prediction of disease risk, XGBoost model, traditional machine learning, comparative analysis

## Abstract

In view of the harm of diabetes to the population, we have introduced an ensemble learning algorithm—EXtreme Gradient Boosting (XGBoost) to predict the risk of type 2 diabetes and compared it with Support Vector Machines (SVM), the Random Forest (RF) and K-Nearest Neighbor (K-NN) algorithm in order to improve the prediction effect of existing models. The combination of convenient sampling and snowball sampling in Xicheng District, Beijing was used to conduct a questionnaire survey on the personal data, eating habits, exercise status and family medical history of 380 middle-aged and elderly people. Then, we trained the models and obtained the disease risk index for each sample with 10-fold cross-validation. Experiments were made to compare the commonly used machine learning algorithms mentioned above and we found that XGBoost had the best prediction effect, with an average accuracy of 0.8909 and the area under the receiver’s working characteristic curve (AUC) was 0.9182. Therefore, due to the superiority of its architecture, XGBoost has more outstanding prediction accuracy and generalization ability than existing algorithms in predicting the risk of type 2 diabetes, which is conducive to the intelligent prevention and control of diabetes in the future.

## 1. Introduction

Diabetes is a clinical syndrome that interacts with environmental and genetic factors and it is also a typical chronic disease from which middle-aged and elderly people suffer a lot. Diabetes is characterized by high blood sugar, with many complications such as microvascular complications, large vascular complications and neurological complications that are really common. For example, microvascular complications are able to cause diabetic eye lesions, resulting in blurred vision, blindness, diabetic retinopathy and diabetic nephropathy. As for diabetic nephropathy, the proteinuria appears first, later the kidney function gradually decreases and eventually kidney failure will show up and develop into uremia, which badly harms human health. According to the recent research [1,2], more than half of middle-aged and elderly people don’t have a strong prevention awareness of the disease, and have no concept of self-management behavior for diabetes. Therefore, establishing a sound warning system for the risk of diabetes will provide portable help for the middle-aged and elderly people to prevent diabetes and pay more attention to it, which is of great significance to reduce the incidence of diabetes in China [3].

Statistical models are widely applied in the early warning of traditional chronic disease risk, such as multilinear regression and multi-logistic regression, which are more often used [4]. The method used a regression coefficient to indicate the correlation degree between risk factors and disease, but its algorithm was relatively single and the results couldn’t accurately give specific probability values. At present, with the development of artificial intelligence, machine learning is progressively used to assess the risk of chronic diseases. It summarizes and learns the factors associated with chronic diseases, and ultimately reaches an evaluation score on possible outcomes of the disease. For example, Finkelstein et al. [5] predicted asthma attacks using Naive Bayes classifiers, adaptive Bayes networks, and Support Vector Machines (SVM), with a sensitivity of 0.80, 1.00 and 0.84; a specificity of 0.77, 1.00 and 0.80, and an accuracy of 0.77, 1.00 and 0.80. Meanwhile, similar studies have been conducted in the field of diabetes prediction. In recent years, Park et al. [6] applied reverse propagation learning sequential multi-layer perceptron (SMLP) to obtain the prediction probability of diabetes, finding that the results were superior to regression models. Zhu et al. [7] improved the logistic regression algorithm intelligently, and the accuracy improved by 1.98% in diabetes prediction. In addition, Sudharsan et al. [8] used machine learning models to predict hypoglycemia events occurring in 24 h in diabetics, resulting in a sensitivity of 92% and a specificity of 70%. However, the prediction of diabetes through machine learning is mainly based on traditional classification algorithms in current research, but reports on the powerful Boosting algorithm application in this area are relatively scarce.

In view of the lack of embodiment of novel and efficient ensemble learning in the study of evaluating type 2 diabetes risk, combined with its advantages in the field of data mining, we built a EXtreme Gradient Boosting (XGBoost) model [9,10,11,12] to predict the risk of diabetes among 368 middle-aged and elderly people, and finally drew the specific probability of each potential patient. To our knowledge, this was an innovative application of the XGBoost algorithm in this field. At the same time, in order to reflect the superiority of this method, we also compared it with the traditional machine learning models in diabetes risk assessment. This study indicates that despite the small amount of data used for training, the XGBoost model still exhibits stable and excellent performance.

## 2. Materials and Methods

**Figure 1:**
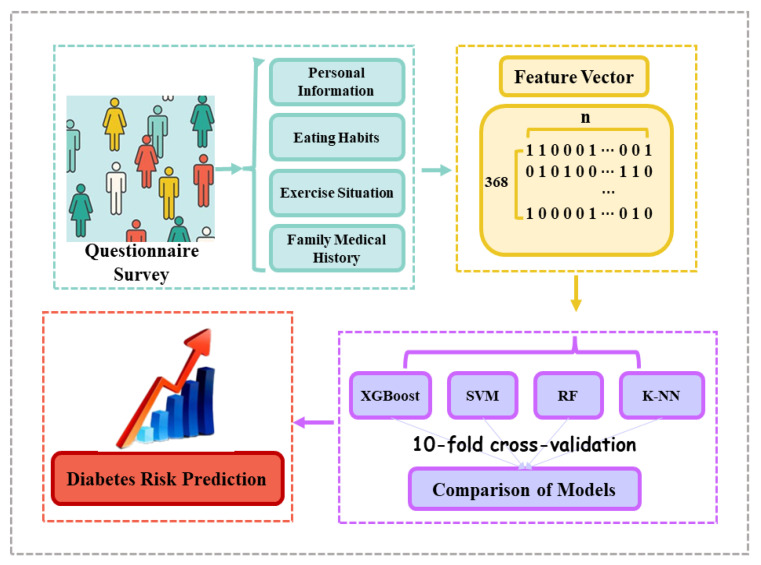
The workflow of this research.

The workflow of this paper is shown in Figure 1. The collected questionnaires were converted into feature vectors in the form of binary codes after strict selection. Meanwhile, the research used 4 machine learning models (XGBoost, SVM, Random Forest (RF) and K-Nearest Neighbor (K-NN)) to conduct experiments, and evaluated the performance of various algorithms through 10-fold cross-validation. Ultimately, we chose the optimal model to predict the risk of type 2 diabetes.

### 2.1. Experimental Data

A questionnaire survey that combined convenient sampling with snowball sampling was conducted in the Xicheng district of Beijing, and the main target population was the middle-aged (between 45 and 54 years old) and elderly people (55 years and older). In our research, location and time period of the survey, age, gender and illness of the respondents were chosen randomly. The survey content can be divided into 4 categories of information, namely, personal information, eating habits, exercise situation and family history, whereby each of them has multiple problems. Each question except personal information and family history took the frequency as choices, including 3 times a day and above, 2 times a day, 1 time a day, 4–6 times a week, 1–3 times a week, 1–3 times per month and never. A total of 380 questionnaires were distributed in the survey, and 368 valid questionnaires were eventually obtained after the data was cleaned.

It is worth noting that the type 2 diabetes prevalence of each respondent had been strictly confirmed (referring to the World Health Organization standard, FPG greater than or equal to 7.0 mmol/L is considered as diabetes). In addition, the questionnaire information filled by the investigators had been strictly confirmed to prevent human errors caused by the investigators’ own reasons.

It must be emphasized that no human subjects were used in this study, we only adopted their personal information for analysis with the permission of the questionnaire subjects. Meanwhile, this work was approved by the Human Research Ethics Committee in China Agricultural University (approval number: CAUHR-2020003).

### 2.2. Feature Vectors Representation

We needed to convert the questionnaire information into feature vectors that could be input into the machine learning models. Here, a feature representation method based on binary coding was provided. For personal information and family history, the experiment converted the yes/no answer of each question (such as whether there is a family history, etc.) into a number 1/0. In addition, eating habits and exercise situation were another way. More specifically, for the K questions on eating habits, each question had 7 options, assuming that a patient met the first option, the expression was *f*(*q_1_*) = (1, 0,…, 0); if it matched the second option, the expression was *f*(*q_1_*) = (0, 1,…, 0), and so on. In the same way, for the M questions about exercise situations, the expression was similar. The corresponding formula is as follows.

(1) $$F=\left[f(q_{1}),f\left(q_{2}\right),\ldots ,f\left(q_{k}\right)\right]$$

where *F* represents the feature vector of each sample’s eating habits and exercise situation. The total feature vector also requires coding of personal information and family history.

### 2.3. EXtreme Gradient Boosting Algorithm

First, the answers to all questions in each sample were converted to a feature vector, which acted as an input vector of the XGBoost model. This article was programmed with Python 3.8 and was modeled and trained on the configuration of the Windows 10 (Microsoft, Redmond, WA, USA) Operating System, and the CPU was Intel Core I7-6700HQ, 3.5 GHz, with a memory of 4 GB.

XGBoost is a novel machine learning algorithm that was born in February 2014. This algorithm has gained wide attention because of its excellent learning effect and efficient training speed. The XGBoost algorithm is an improvement of the gradient boosting decision tree (GBDT) and can be used for both classification and regression problems. It is worth noting that XGBoost is also one of the boosting tree algorithms, which is to integrate many weak classifiers together to form a strong classifier. The tree model it uses is the classification and regression tree (CART) model.

The idea of this algorithm is to add trees continuously, and to split the features continuously to grow a tree. Each time you add a tree, you actually learn a new function to fit the last predicted residual. When we finish training to get *k* trees, we have to predict the score of a sample. In fact, according to the characteristics of this sample, a corresponding leaf node will fall in each tree, and each leaf node corresponds to a score. The score corresponding to each tree needs to be added up to be the predicted value of the sample. Specifically, the workflow of the algorithm is as follows.

1. Before starting to iterate the new tree, calculate the first and second derivative matrices of the loss function corresponding to each sample.
2. Each iteration adds a new tree, and each tree fits the residual of the previous tree.
3. Count the split gain value of the objective function to select the best split point, and employ the greedy algorithm to determine the best structure of the tree.
4. Add a new tree to the model and multiply it by a factor to prevent overfitting. When fitting residuals, step size or learning rate are usually used to control optimization, so as to reserve more optimization space for subsequent learning.
5. After training, a model of multiple trees is obtained, in which each tree has multiple leaf nodes.
6. In each tree, the sample falls on several leaf nodes according to the eigenvalues. The final predicted value is the score of the leaf node corresponding to each tree multiplied by the weight of the tree.

From the perspective of model expression, suppose we iterate *t* rounds, which means that we want to generate *t* residual trees. At this time, the expression of the model prediction value is as follows:

(2) $${\hat{y}}_{i}=\sum_{t=1}^{T}f_{t}\left(x_{i}\right),f_{t}\in F$$

where *f_t_(x_i_)* represents the predicted value of the t-th residual tree for the t-th residual of *x_i_*. *y_i_* represents the predicted value of the model, *f_t_* is the residual number of the t-th round, and *F* is the function space of the residual tree. Additionally, the loss function is also an indispensable part of this algorithm, and its error sources are mainly: training errors and model complexity. Its main formula is as follows:

(3) $$obj=\sum_{i=1}^{n}l\left(y_{i,}{\hat{y}}_{i}\right)+\sum_{k=1}^{K}\Omega \left(f_{k}\right)$$

where *l* represents the loss function and Ω is the regularization term. For the t-th round of training, the above loss expression satisfies the following relationship:

(4) $${\hat{y}}_{i}^{t}={\hat{y}}_{i}^{t-1}+f_{i}\left(x_{i}\right)$$

(5) $$\Omega \left(f_{t}\right)=\left(\sum_{k=1}^{T-1}\Omega \left(f_{k}\right)\right)+\Omega \left(f_{t}\right)=const+\Omega \left(f_{t}\right)$$

Therefore, the loss of the t-th round combined with the second-order Taylor expansion can be simplified into the following form:

(6) $$\begin{array}{l} obj^{t}\approx \sum_{i=1}^{n}\left[l\left(y_{i},{\hat{y}}_{i}^{t-1}\right)+g_{i}f_{i}\left(x_{i}\right)+\frac{1}{2}h_{i}{\left(f_{t}\left(x_{i}\right)\right)}^{2}\right]+\Omega \left(f_{t}\right)+const\\ =\sum_{i=1}^{n}\left[g_{i}f_{t}\left(x_{i}\right)+\frac{1}{2}h_{i}\left(f_{t}\left(x_{i}\right)\right){}^{2}\right]+\Omega \left(f_{t}\right)+const \end{array}$$

In this objective function, *g_i_* and *h_i_* are the parameters of the t-th residual tree, so the minimum value of the function can be obtained only by determining the parameters according to the number of leaf nodes. It should also be emphasized that the optimal result of the model also depends on the structure of the tree, and XGBoost adopts the greedy algorithm to generate the specific architecture of the tree. Specifically, the algorithm starts at the root node and traverses all features. For each feature, if it is a continuous feature, it is arranged from small to large. There are 368 samples in this experiment, so there are 367 split points for this continuous feature, and a split gain value is calculated at each split point. This value is employed to determine whether the current node needs to be split and to select the best split point.

In our experiments, XGBoost was implemented in the scikit-learn machine learning library under Python 3.8. We optimized the parameters of the model during the 10-fold cross-validation.

### 2.4. Baseline Algorithms

In order to reflect the superiority of XGBoost in the field of diabetes risk prediction, three commonly used chronic disease prediction algorithms (SVM, RF, K-NN) were selected to make a comparison with the methods above. The training process was made in Python 3.8 with 10 fold cross-validation, and the parameters was adjusted to a relatively high level.

Specifically, this paper designed a nonlinear SVM model that performed a binary classification task, and its kernel function was RBF. SVM can easily obtain the non-linear relationship between data and features when the sample size is small. It can avoid the use of neural network structure selection and local minimum problems. It has strong interpretability and can solve high-dimensional problems.

Random forest was employed as a classic ensemble learning algorithm to compare with XGBoost. In this experiment, the Bootstraping method was used to randomly select a certain sample from the original training set. A total of n_tree = 20 samples were sampled to generate n_tree = 20 training sets. Each split of each decision tree model was based on information gain to select the best feature. Each tree had been split in this way until all the training examples of the node belonged to the same class. We decided the final classification result according to the votes of multiple tree classifiers.

Moreover, we adopted the K-NN algorithm to calculate the distance between the new data and the feature values of the training data, and then selected K (K ≥ 1) nearest neighbors for classification or regression. If K = 1, then the new data would be assigned to its neighbor class. After continuous experiments, it was found that the model performed best when K = 5.

### 2.5. Parameters Adjustment

The experiment employed the GridSearchCV method for automatic parameters adjustment. We used this method to select the optimal parameters of the above 4 models. The final key parameters are shown in Table 1, Table 2, Table 3 and Table 4. It must be emphasized that GridSearchCV is only suitable for smaller datasets, while large datasets will have problems such as time-consuming training and slow search.

### 2.6. Statistical Analysis

After training the model above, we predicted the probability of diabetes disease and made a comparation and evaluation for the models through 10-fold cross validation. The evaluation indicators were based on accuracy (Acc), sensitivity (Sens), specificity (Spec), precision (Prec), and Matthew correlation coefficient t (MCC) (the corresponding calculation formulas are as follows). In addition, the Receiver Operating Characteristics (ROC) curves and the AUC values were also taken into account.

(7) $$\mathrm{Acc}=\frac{TN+TP}{TN+TP+FN+FP}$$

(8) $$\mathrm{Sens}=\frac{TP}{TP+FN}$$

(9) $$\mathrm{Spec}=\frac{TN}{TN+FP}$$

(10) $$\mathrm{Prec}=\frac{TP}{TP+FP}$$

(11) $$\mathrm{MCC}=\frac{TP\times TN-FP\times FN}{\sqrt{\left(TP+FP)(TP+FN)(TN+FP)(TN+FN\right)}}$$

wherein, *TP* (true positive) is the proportion of the positive samples correctly classified, *FP* (false positive) is the proportion of samples shown correctly classified as belonging to a specific class when they actually do not belong to that class, *TN* (true negative) is the proportion of the negative samples correctly classified and *FN* (false negative) represents the number of samples classified as not belonging to a specific class when they actually do belong to that class.

## 3. Results

### 3.1. Statistics Results

We have collected 380 samples in total during this survey; after removing 12 cases that were missing and obviously inconsistent, there were 368 samples left for analysis. Table 5 counts the detailed information of the questionnaire, including the investigator’s personal information, eating habits, exercise situation, and family medical history, and summarizes the number and proportion of respondents at each point.

### 3.2. Training and Testing Sets Distribution

In this paper, 10-fold cross validation method was employed, that is, the dataset was divided into 10 parts, 9 of which were taken in turn as the training set, 1 as the test set, and the average value of the 10 results was used as the evaluation value of the algorithm performance. Meanwhile, the experiment repeated the above process 10 times and 10 evaluation values were obtained for each model, and their mean values and corresponding 95% confidence intervals were counted. Additionally, the whole dataset had 108 experimental positive samples and 260 negative samples. Positive and negative samples were proportionally divided into training and test sets during cross-validation.

### 3.3. Models Comparison

#### 3.3.1. XGBoost Model Results

After adjusting parameters, the XGBoost model achieved relatively excellent performance through 10-fold cross validation, with a training accuracy of 0.9492. Then, we evaluated the results of 10 cross-validations, and the test found that the model had a good predictive effect on the diabetes disease of 37 random potential patients (randomly assigned test set during cross-validation). The average prediction accuracy was 0.8909, the sensitivity was 0.9388, the specificity was 0.7571, the precision was 0.7944, the Matthew correlation coefficient was 0.6589 and the AUC value was 0.9182.

#### 3.3.2. Comparison with Baseline

**Figure 2:**
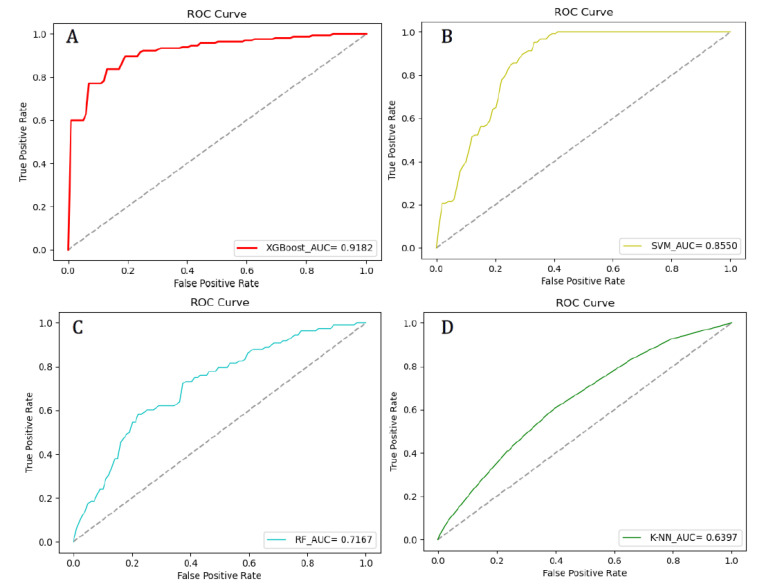
Receiver Operating Characteristics (ROC) curves for each model. (**A**) is the ROC curve of the XGBoost model; (**B**) is the ROC curve of the SVM model; (**C**) is the ROC curve of the RF model; (**D**) is the ROC curve of the K-NN model.

The experiment also compared the proposed model with the baseline models (SVM, RF, K-NN). The parameters were well adjusted and the average value of the 10 test results were used as the final model evaluation value, as shown in Table 6. It is not difficult to find that traditional machine learning algorithms were not as good as XGBoost algorithms in terms of prediction accuracy or AUC values. Among traditional algorithms, SVM had the highest accuracy (0.8158), highest specificity (0.6364), highest precision (0.8111) and highest Matthew correlation coefficient (0.5410) while K-NN had the highest sensitivity (0.9630). Additionally, SVM (0.8550) had the highest AUC value among traditional machine learning, but it was lower than the AUC value of XGBoost (0.9182). The ROC curves of each model are as shown in Figure 2.

### 3.4. Diabetes Risk Prediction

In view of the accuracy of XGBoost in the prediction of type 2 diabetes risk, we counted the predicted probability values of the test set during the cross-validation process after the model was trained. That is, using the ensemble learning model can determine the probability of positive and negative samples of unknown data. It can also provide portable, intelligent self-management and early warning for the vast number of middle-aged and elderly groups in the future, which is conducive to preventing the occurrence of diabetes reducing the incidence of diabetes.

## 4. Discussion

Previous studies have shown that logistic regression models are often applied in single-element or multi-element diabetes risk assessment, such as Abdullah et al. [13] making use of environmental and genetic risk factors to predict the risk of type 2 diabetes in Malaysia with a fitted multiple logistic regression model and as a result, the AUC value was 0.75–0.83. However, the logistic regression model can’t both carry out feature study of sample data and obtain the prediction results of unknown samples, which means it has great limitations. With the development of artificial intelligence, many machine learning models have been employed in the research of predicting the risk of diabetes early. The most commonly used is the SVM supervision model. It selects the feature space to construct the optimal superplane on the basis of the summary of structural risk minimization theory, enabling the classifier to obtain global optimization, and meeting a certain upper bound with a certain probability value in the sample space expectation. Previous studies have shown that SVM has a strong generalization ability and performed excellently on many forecasting issues [14,15,16]. SVM is also widely used in the field of diabetes prediction, for example, Xiong et al. [17] utilized machine learning algorithms to build a prediction model of type 2 diabetes, finding the result was really satisfied. In our research, SVM performed best in traditional machine learning algorithms, with a sensitivity of 0.8889 and an AUC value of 0.8550. RF is a classic Bagging integrated learning algorithm, which can handle higher dimensional data while and reducing the impact of unbalanced data at the same time, but sometimes it is easily over-fitting when it comes to the classification issues of over-loud noise. Meanwhile, RF is widely used in the field of diabetes risk prediction [18,19]. In our experiment, RF didn’t perform as well as SVM after integration; the reason may be situated in the poor classification effect when processing small amounts of data or low-dimensional data. K-NN is another commonly used algorithm in diabetes prediction research with a simple algorithm, unsophisticated training process and weak interpretability, but it has a low accuracy of prediction for rare categories when the sample is unbalanced. The type 2 diabetes data set in our study was unbalanced and the number of positive samples was relatively small, so the sensitivity of the experiment was high (0.9630) while the specificity was extremely low (0.1818). Therefore, the sample could not be well predicted.

In this paper, a novel ensemble learning algorithm was used to predict the risk of type 2 diabetes, and this is a successful example of XGBoost’s application in the study of chronic disease risk assessment. We believe this may be inseparable from the superiority of the algorithm itself. XGBoost is an ensemble learning algorithm, which belongs to the boosting algorithm category in three commonly used ensemble methods (bagging, boosting, stacking). It is an addition model, and its base learner can be either a CART model or a linear classifier [20]. Specifically, XGBoost is improved by the GBDT algorithm. Its basic idea is to fit the deviation of the previous model through the new base model, thereby continuously reducing the deviation of the addition model. In this experiment, it has the following advantages. First of all, XGBoost adds regularization terms to the objective function, which avoids overfitting the model due to insufficient sample size in diabetic patients. Second, the algorithm takes into account the fact that the training data is sparse. You can specify the default direction of the branch for missing values or specified values, which can greatly improve the efficiency of the algorithm. This advantage reduces the difficulty of training. Moreover, it also borrows from the practice of random forests—it supports column sampling, which can not only reduce overfitting, but also reduce calculations. Compared with the simplicity and singularity of traditional algorithm architecture, XGBoost has the advantage of principle, which made it stand out in the prediction of type 2 diabetes risk.

Although the XGBoost model performed better than the traditional algorithm in this paper, it is undeniable that it adopts the pre-sorting principle. Before the iteration, the model pre-sorts the characteristics of the nodes and traverses to select the optimal split point. This results in finding that the optimal greedy algorithm takes a long time and the training difficulty increases when the amount of data is large. Therefore, the XGBoost algorithm is more suitable for small sample training similar to this task.

Our research also has certain flaws, which deserve to be improved in the future. Firstly, the experimental sample size should be further expanded so that more reliable conclusions can be drawn. Secondly, the patient information input into the model needs to be further improved, because only comprehensive information can predict type 2 diabetes more accurately. Thirdly, because machine learning has a “black box” feature, we cannot explain how it works at the moment. Future efforts in this direction will be required. In addition, cross-sectional data has inherent defects, and large-scale prospective or retrospective research will continue to be carried out in the future.

## 5. Conclusions

The study aimed at working out the problem that the prediction accuracy and the generalization performance of models are not satisfied in the field of predicting the risk of suffering from diabetes. Our study made use of the XGBoost model, one of the ensemble learning algorithms, to make a disease risk assessment of type 2 diabetes for potential patients, and then compared it with the current mainstream algorithms (SVM, RF and K-NN). The prevalence of random samples were predicted based on the personal information, eating habits, exercise condition and family history of diabetes of the middle-aged and elderly people surveyed, and then calculated the index of suffering from diabetes. The results showed that XGBoost has the strongest generalization ability and prediction accuracy, which can better estimate the risk of potential patients and provides new ideas for intelligent prevention of chronic diseases in the future.

## Figures and Tables

**Table 1:** Key parameters of the EXtreme Gradient Boosting (XGBoost) model.

Parameter Name	Value
gamma	0.1
max_depth	0.5
lambda	3.0
subsample	0.7
silent	1.0
eta	0.1
seed	1000.0

**Table 2:** Key parameters of the Support Vector Machines (SVM) model.

Parameter Name	Value
C	1.0
degree	3.0
epsilon	0.2
gamma	auto
tol	0.001

**Table 3:** Key parameters of the Random Forest (RF) model.

Parameter Name	Value
n_estimators	60
max_depth	13
max_features	9
Random_state	20
oob_score	True

**Table 4:** Key parameters of the K-Nearest Neighbor (K-NN) model.

Parameter Name	Value
n_neighbors	5
n_jobs	1

**Table 5:** Detailed statistics of the questionnaire information.

Type of Information		Classification	Number of People (Individual)	Proportion (%)
Personal Information	Education Level	High Educational Background	237	64.40
		High School Degree or Even Lower	131	35.60
	Marriage	Married	304	82.61
		Unmarried	64	17.39
Eating Habits	Rice	Three Times a Day or Even More	324	88.04
		Twice a Day	20	5.43
		Once a Day or Even Not	24	6.52
	Buns	Three Times a Day or Even More	312	84.78
		Twice a Day	32	8.70
		Once a Day or Even Not	24	6.52
	Noodles	Three Times a Day or Even More	38	10.33
		Twice a Day	67	18.21
		Once a Day or Even Not	263	71.47
	Pork	Three Times a Day or Even More	156	42.39
		Twice a Day	127	34.51
		Once a Day or Even Not	85	23.10
	Beef	Three Times a Day or Even More	120	32.61
		Twice a Day	93	25.27
		Once a Day or Even Not	155	42.12
	Fish	Three Times a Day or Even More	145	39.40
		Twice a Day	129	35.05
		Once a Day or Even Not	94	25.54
	Seafood	Three Times a Day or Even More	31	8.42
		Twice a Day	54	14.67
		Once a Day or Even Not	283	76.90
	Dairy Products	Three Times a Day or Even More	62	16.85
		Twice a Day	88	23.91
		Once a Day or Even Not	218	59.24
	Fruits	Three Times a Day or Even More	89	24.18
		Twice a Day	172	46.74
		Once a Day or Even Not	107	29.08
	Vegetables	Three Times a Day or Even More	217	58.97
		Twice a Day	102	27.72
		Once a Day or Even Not	49	13.32
Exercise Situation	Leisure Sports	Twice a Day or Even More	102	27.72
		Once a Day	132	35.87
		4 To 6 Times a Week or Even Less	134	36.41
	Vigorous Sports	Twice a Day or Even More	22	5.98
		Once a Day	43	11.68
		4 To 6 Times a Week or Even Less	303	82.34
Family Medical History		Have	101	27.45
		Don’t Have	267	72.55

**Table 6:** Comparison of effects for each model.

Machine Learning Model	Evaluation Indicators					
	Acc	Sens	Spec	Prec	MCC	AUC
XGBoost	0.8909 ± 0.0177	0.9388 ± 0.0251	0.7571 ± 0.0405	0.7944 ± 0.0296	0.6589 ± 0.0537	0.9182 ± 0.0130
SVM	0.8158 ± 0.0112	0.8889 ± 0.0255	0.6364 ± 0.0239	0.8571 ± 0.0227	0.5410 ± 0.0650	0.8550 ± 0.0479
RF	0.7895 ± 0.0308	0.9259 ± 0.0219	0.4545 ± 0.0328	0.8065 ± 0.0365	0.4451 ± 0.0921	0.7167 ± 0.0356
K-NN	0.7368 ± 0.0225	0.9630 ± 0.0199	0.1818 ± 0.0240	0.7429 ± 0.0294	0.2548 ± 0.0554	0.6397 ± 0.0295

Notes: the values represent average values and their 95% confidence intervals.
